# Piezo1 channel activation mimics high glucose as a stimulator of insulin release

**DOI:** 10.1038/s41598-019-51518-w

**Published:** 2019-11-14

**Authors:** Vijayalakshmi Deivasikamani, Savitha Dhayalan, Yilizila Abudushalamu, Romana Mughal, Asjad Visnagri, Kevin Cuthbertson, Jason L. Scragg, Tim S. Munsey, Hema Viswambharan, Katsuhiko Muraki, Richard Foster, Asipu Sivaprasadarao, Mark T. Kearney, David J. Beech, Piruthivi Sukumar

**Affiliations:** 1 Leeds Institute for Cardiovascular and Metabolic Medicine, Faculty of Medicine and Health, University of Leeds, Leeds, LS2 9JT United Kingdom; 2 School of Chemistry, Faculty of Mathematics and Physical Sciences, University of Leeds, Leeds, LS2 9JT United Kingdom; 3 School of Biomedical Sciences, Faculty of Biological Sciences, University of Leeds, Leeds, LS2 9JT United Kingdom; 4 School of Pharmacy, Aichi-Gakuin University, 1-100 Kusumoto, Chikusa, Nagoya 464-8650 Japan

**Keywords:** Gastrointestinal hormones, Intracellular recording, Cell biology, Endocrine system and metabolic diseases

## Abstract

Glucose and hypotonicity induced cell swelling stimulate insulin release from pancreatic β-cells but the mechanisms are poorly understood. Recently, Piezo1 was identified as a mechanically-activated nonselective Ca^2+^ permeable cationic channel in a range of mammalian cells. As cell swelling induced insulin release could be through stimulation of Ca^2+^ permeable stretch activated channels, we hypothesised a role for Piezo1 in cell swelling induced insulin release. Two rat β-cell lines (INS-1 and BRIN-BD11) and freshly-isolated mouse pancreatic islets were studied. Intracellular Ca^2+^ measurements were performed using the fura-2 Ca^2+^ indicator dye and ionic current was recorded by whole cell patch-clamp. Piezo1 agonist Yoda1, a competitive antagonist of Yoda1 (Dooku1) and an inactive analogue of Yoda1 (2e) were used as chemical probes. Piezo1 mRNA and insulin secretion were measured by RT-PCR and ELISA respectively. Piezo1 mRNA was detected in both β-cell lines and mouse islets. Yoda1 evoked Ca^2+^ entry was inhibited by Yoda1 antagonist Dooku1 as well as other Piezo1 inhibitors gadolinium and ruthenium red, and not mimicked by 2e. Yoda1, but not 2e, stimulated Dooku1-sensitive insulin release from β-cells and pancreatic islets. Hypotonicity and high glucose increased intracellular Ca^2+^ and enhanced Yoda1 Ca^2+^ influx responses. Yoda1 and hypotonicity induced insulin release were significantly inhibited by Piezo1 specific siRNA. Pancreatic islets from mice with haploinsufficiency of Piezo1 released less insulin upon exposure to Yoda1. The data show that Piezo1 channel agonist induces insulin release from β-cell lines and mouse pancreatic islets suggesting a role for Piezo1 in cell swelling induced insulin release. Hence Piezo1 agonists have the potential to be used as enhancers of insulin release.

## Introduction

Secretion of insulin by pancreatic β-cells is vital for maintaining blood glucose and energy homeostasis. Although insulin biosynthesis is controlled by multiple factors, glucose metabolism is the most important physiological event that stimulates insulin secretion^1^. Physiologically, glucose enters the β-cells through an insulin independent process (involving glucose transporters (GLUT); GLUT1 and 3 in humans and GLUT2 in rodents^2^) where it undergoes glycolysis, enters the TCA cycle and results in the generation of ATP. An increased ATP/ADP ratio leads to the closure of ATP-dependent potassium (K_ATP_) channels which induces membrane depolarisation which in turn, activates voltage-dependent Ca^2+^ channels (VDCC), leading to influx of Ca^2+^. Elevated intracellular Ca^2+^ forms the trigger for insulin release. Insulin is then released into the circulation by the fusion of granules with the cell membrane and exocytosis^3^. Apart from the conventional ATP-K_ATP_-VDCC pathway of insulin release in response to glucose stimulation, osmotic cell swelling resulting from increased glucose levels or hypotonicity has also been reported to induce insulin secretion^4–7^. Glucose induced cell swelling is suggested to be due to lactate accumulation and/or activation of Na^+^/H^+^ and Cl^−^/HCO_3_ exchangers^8^. Such insulin secretion was not completely inhibited by K_ATP_ blockers^8,9^. Cell swelling has been suggested to stimulate insulin release by activating anionic channels in pancreatic β-cells, namely the volume sensitive chloride (Cl^−^) channels, thus depolarizing the cell membrane and increasing the cytosolic Ca^2+^ concentration through activation of VDCC^4,5,10,11^. However, some investigators have argued against this idea as hypotonically induced insulin secretion persisted even in the presence of Cl^−^ channel blockers such as niflumic acid and DIDS^7,12^. Thus it is possible that a mechanism independent of Cl^−^ channels is involved in hypotonically induced insulin secretion. Moreover, a study on rat pancreatic islets *in vitro* has revealed that glucose and hypo-osmotic cell swelling induce insulin secretion through distinct signal transduction pathways^13^. Osmotic cell swelling mechanically stretches the plasma membrane and hence the role of stretch activated cation channels was investigated^14^. The latter study revealed that hypotonicity induced osmotic cell swelling in rat pancreatic β-cells results in the activation of certain stretch activated cationic channels which results in Ca^2+^ entry and insulin release. However, no report currently exists on the identity of stretch activated channels in pancreatic β-cells^8^.

Recently, a novel type of stretch activated Ca^2+^ permeable non selective cationic channels named Piezo1 has been identified in various mammalian cells^15^. Piezo1 is expressed in the lungs, bladder, kidney, endothelial cells, erythrocytes, skin, periodontal ligament cells and elsewhere^16^. While no reports exists on the presence of Piezo1 in insulin secreting β-cells, Piezo1 of pancreatic acinar cells is implicated in pancreatitis^17^. Pancreatic transcriptome databases suggest expression of Piezo1 in primary α-cells and β-cells^18,19^. Piezo1 channels are responsible for mechanically activated cationic currents in numerous eukaryotic cell types, converting mechanical forces to biological signals^20^. Piezo1 has been reported to play a significant role in endothelial cell biology, collecting duct osmoregulation, urothelial pressure sensing, cell migration and erythrocyte volume regulation^21–25^. Piezo1 is a large integral membrane protein with 38 transmembrane segments which assemble as trimers to form ion channels^26,27^. Piezo1 can be specifically activated by Yoda1, a key compound used to study Piezo1 regulation and function, and is inhibited by common non-specific small molecule inhibitors such as ruthenium red (RR) and gadolinium ions (Gd^3+^)^28,29^. Recently, a Yoda1 analogue named Dooku1 has been identified which antagonizes Yoda1 activation of Piezo1^30^.

We hypothesised that Piezo1 could contribute to stretch induced Ca^2+^ influx caused by cell swelling in β-cells, and thereby regulate insulin secretion. In order to test our hypothesis, two rat β-cell line models (INS-1 and BRIN-BD11) and mouse primary pancreatic islets were used in conjunction with chemical modulators of Piezo1. Our results indicate that Piezo1 channels have a significant role in hypotonicity/cell swelling induced insulin release from β-cells.

## Results

### INS-1 cells express Piezo1 and respond to Piezo1 agonist

**Figure 1:**
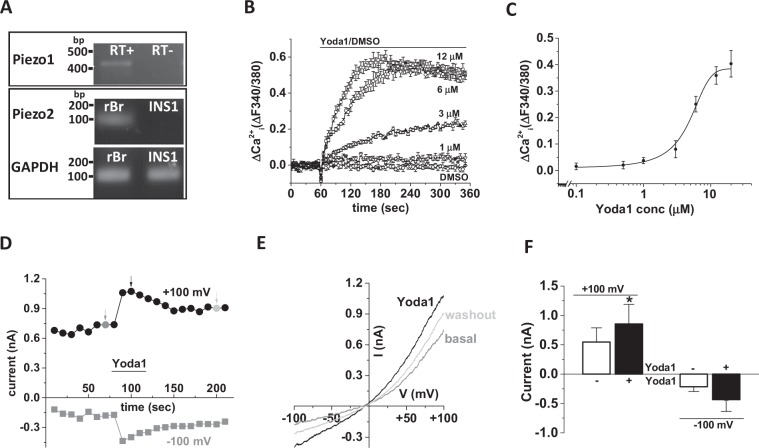
INS-1 cells express functional Piezo1 channels. (**A**) Representative agarose gel electrophoresis picture showing 473 bp band for Piezo1 mRNA expression and no band for Piezo2 (RT: with (+) and without (−) reverse transcriptase; bp: base pairs;). Rat brain (rBr) was used as positive control for Piezo2 (102 bp) and GAPDH (106 bp) expression was used as positive control for the RT-PCR. (**B)** Example data showing Yoda1 (increasing doses) or DMSO (control) induced Ca^2+^ influx in INS-1 cells. (**C)** Mean data obtained from 5 independent experiments of the type shown in (**B**) showing the concentration-response curve for Yoda1 effect. (**D,E**) Representative time-series (**D**) and current-voltage relationships (**E**) showing 5 μM Yoda1 induced current in INS-1 cells in whole cell patch-clamp recording. (**F)** Mean current at +100 mV and −100 mV with and without Yoda1 in INS-1 cells (n = 6–7).

**Figure 2:**
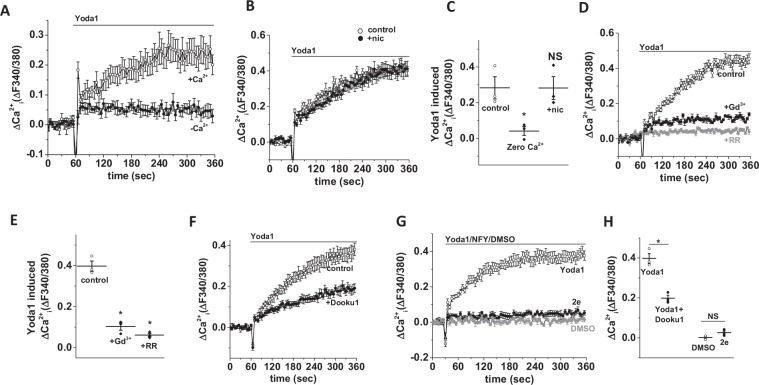
Characteristics of Yoda1-induced Ca^2+^ influx in INS-1 cells. (**A**,**B**) Example data showing the effect of zero extracellular Ca^2+^ (**A**) and nicardipine (**B**) pre-treatment on the Yoda1-mediated Ca^2+^ response in INS-1 cells. (**C**) Mean data obtained from 3 independent experiments of the types shown in (**A,B**). (**D**) Example data showing the effect of gadolinium (Gd^3+^) and ruthenium red (RR) pre-treatment on Yoda1-mediated Ca^2+^ response in INS-1 cells. **E)** Mean data obtained from 3 independent experiments of the types shown in (**D**). (**F**) Example data showing the effect of Dooku1 pre-treatment on the Yoda1-mediated Ca^2+^ response in INS-1 cells. (**G**) Example data showing the effect of Yoda1, a non-functional Yoda1 analogue (2e) and DMSO (control) on Ca^2+^ influx in INS-1 cells. (**H**) Mean data obtained from 3 independent experiments of the types shown in (**F**,**G**). Data are expressed as mean ± sem; * denotes p < 0.05 vs control.

Piezo1 mRNA, but not Piezo2 mRNA was detected in INS-1 cells by RT-PCR (Fig. 1A). Anticipating that functional Piezo1 channels are also expressed, the activity of Piezo1 chemical agonist Yoda1 on Ca^2+^ influx was tested on INS-1 cells. In a dose dependent manner Yoda1 induced increases in intracellular Ca^2+^ with an estimated EC_50_ of 4 μM (Fig. 1B,C). Poor solubility of Yoda1 above 10 μM prevented an accurate determination of the EC_50_. We performed voltage-clamp recordings to confirm that Yoda1-induced Piezo1 activation leads to ionic influx in INS-1 cells. Yoda1 evoked ionic currents which had a current-voltage relationship (I-V) consistent with activation of Piezo1 channels (Fig. 1D–F). To investigate if the Ca^2+^ increase depends on influx of Ca^2+^ from the extracellular space, rather than release from intracellular stores, the effect of Yoda1 was tested in the absence of extracellular Ca^2+^. When there was no Ca^2+^ outside, Yoda1 did not increase the intracellular Ca^2+^ signal, indicating that it induces Ca^2+^ influx rather than store release (Fig. 2A,C). Though no Ca^2+^ was added to the extracellular buffer, in the absence of a Ca^2+^ chelator there could be some residual Ca^2+^ extracellularly whose influx might have resulted in the small increase in fluorescence observed. Most β-cell lines including INS-1 cells express VDCC which are essential for the classical insulin secretion pathway. Therefore, cells were pre-treated with the VDCC inhibitor nicardipine (10 μM for 30 min) before the addition of Yoda1. Nicardipine did not affect the Yoda1 stimulation of INS-1 cells, suggesting that the Yoda1 induced Ca^2+^ influx was not through VDCC (Fig. 2B,C). Though Yoda1 is considered to be specific for Piezo1 channels, the risk of non-specific effects must be considered. Thus we have made additional determinations. The non-specific inhibitors Gd^3+^ and ruthenium red (RR), both at 30 μM with 30 min pre-treatment, inhibited Yoda1 induced Ca^2+^ influx (Fig. 2D,E). Moreover, the recently developed Yoda1 analogue (Dooku-1), which antagonises Yoda1 activation of Piezo1, significantly reduced Yoda1 induced Ca^2+^ influx (Fig. 2F,H) and an inactive Yoda1 analogue (2e) failed to mimic the effect of Yoda1 (Fig. 2G,H). These expression and pharmacological data suggest that INS-1 cells contain functional Piezo1 channels.

### Piezo1 agonist response in BRIN-BD11 cells

**Figure 3:**
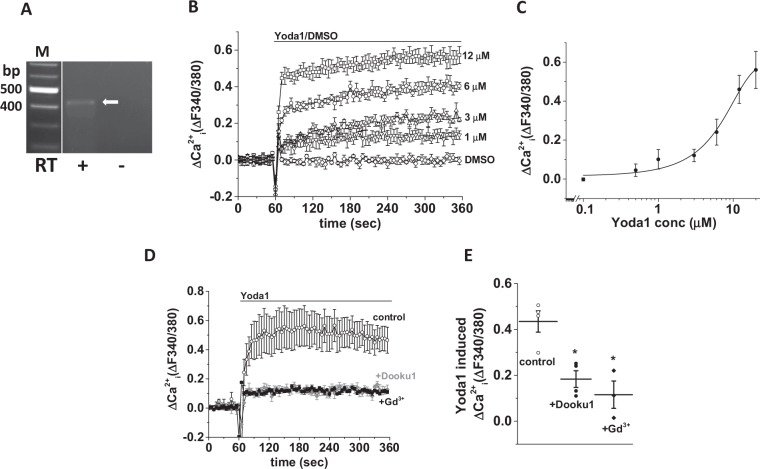
BRIN-BD11 cells express functional Piezo1 channels. (**A**) Representative agarose gel electrophoresis picture with a single band at 473 bp (arrow) showing Piezo1 mRNA expression (RT: with (+) and without (−) reverse transcriptase; bp: base pairs; M: marker). (**B**) Example data showing Yoda1 (increasing doses) or DMSO (control) induced Ca^2+^ influx in BRIN-BD11 cells. (**C**) Mean data obtained from 3 independent experiments of the type shown in (**B**) showing the concentration-response curve for Yoda1 effect. (**D**) Example data showing the effect of gadolinium (Gd^3+^) and Dooku1 pre-treatment on Yoda1-mediated Ca^2+^ response in INS-1 cells. (**E**) Mean data obtained from 3 independent experiments of the types shown in (**D**). Data are expressed as mean ± sem; * denotes p < 0.05 vs control.

To further confirm the expression of functional Piezo1 channels in β-cell lines, we also investigated another β-cell line: BRIN-BD11 cells. These cells also expressed Piezo1 mRNA (Fig. 3A). Similar to INS-1 cells, Yoda-1 induced Ca^2+^ influx occurred in BRIN-BD11 cells (estimated EC_50_ 9 μM; Fig. 3B,C) and was inhibited by Gd^3+^ (30 μM) and Dooku1 (10 μM) (Fig. 3D,E). The data suggest that BRIN-BD11 cells also express functional Piezo1 channels.

### Stimulation of insulin release by osmotic or shear stress

**Figure 4:**
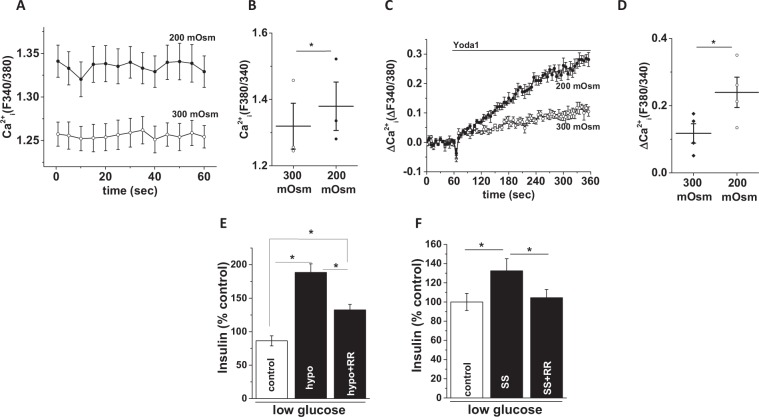
Stimulation of insulin release by osmotic or shear stress. (**A**) Basal Ca^2+^ levels in INS-1 cells in hypotonic (200 mOsm) or isotonic (300 mOsm) extracellular buffer. (**B**) Mean data obtained from 3 independent experiments of the types shown in (**A**). (**C**) Example data showing Yoda1-induced Ca^2+^ influx in INS-1 cells in hypotonic or isotonic buffer. (**D**) Mean data obtained from 3 independent experiments of the types shown in (**C**). (**E**) Mean data showing insulin levels in the extracellular buffer upon treatment of INS-1 cells with hypotonic buffer for 1 hour in the presence or absence of ruthenium red (RR). (**F)** As of (**E**) but showing the effect of shear stress (SS). Insulin levels were measured by ELISA, normalised against controls and expressed as a percentage (%) of control. For RR inhibition experiments, cells were pre-treated with RR for 15 min before exposure to hypotonic buffer or SS. Data are expressed as mean ± sem; * denotes p < 0.05.

An expectation of cells expressing Piezo1 is that they should be sensitive to osmotic stress caused by hypotonic solution and shear stress caused by fluid flow. Hypotonic solution is expected to cause cell swelling because of increased water entry into cells and specifically in β-cells, cell swelling leads to insulin release. Consistent with the presence of functional Piezo1 channels, hypotonicity increased basal and Yoda1 induced intracellular Ca^2+^ levels (Fig. 4A–D). Furthermore, hypotonicity caused insulin secretion which was suppressed by the non-specific Piezo1 channel inhibitor RR (Fig. 4E). Exposing INS-1 cells to the physical force of shear stress (12 dyne/cm^2^ for 1 hour) also induced insulin release and this too was inhibited by RR (Fig. 4F). The data provide further evidence of functional Piezo1 channels in β-cells and suggest a link to insulin secretion. However, it should be noted that RR can inhibit VDCC too which could be secondarily activated by Piezo1 stimulation.

### Piezo1 agonist regulates insulin release from INS-1 cells

**Figure 5:**
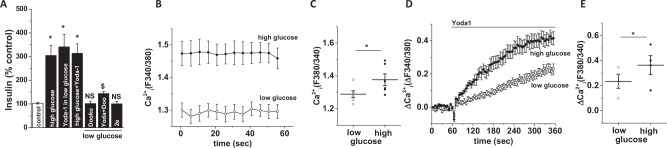
Piezo1 agonist regulates insulin release from INS-1 cells. (**A)** Mean data showing insulin levels in the extracellular solution upon treatment of INS-1 cells with shown chemicals for 1 hour; 2e: non-functional Yoda1 analogue. (**B)** Basal Ca^2+^ levels in INS-1 cells in low glucose (2.8 mM) or high glucose (17.8 mM) containing extracellular buffer. (**C)** Mean data obtained from 3 independent experiments of the types shown in (**B**). (**D)** Example data showing Yoda1 or DMSO (control) induced Ca^2+^ influx in INS-1 cells in low glucose or high glucose containing buffer. (**E)** Mean data obtained from 3 independent experiments of the types shown in (**D**). Data are expressed as mean ± sem; * denotes p < 0.05 vs control; NS: non-significant vs control; $ denotes p < 0.05 vs Yoda1.

**Figure 6:**
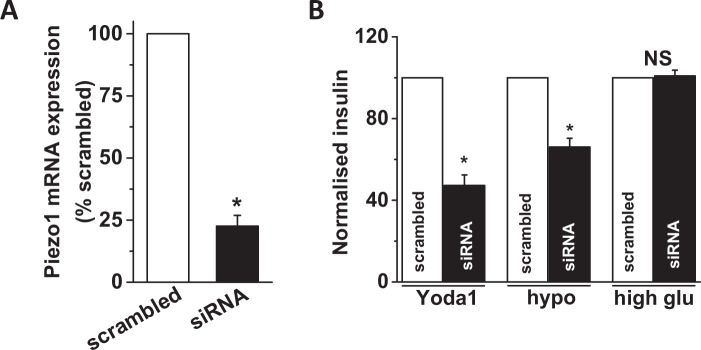
Piezo1 knockdown reduces Yoda1 and hypotonicity induced insulin release from INS-1 cells. (**A)** Mean relative expression of Piezo1 mRNA in INS-1 cells transfected with Piezo1 specific siRNA or scrambled (control) siRNA. (**B)** Mean data showing insulin levels in the extracellular buffer upon treatment of INS-1 cells with Yoda1, hypotonic buffer (hypo) or high glucose (high glu) after transfection with scrambled or Piezo1 siRNA transfection. Insulin levels were normalised against scrambled controls and expressed as a percentage (%) of respective controls. Data are expressed as mean ± sem; * denotes p < 0.05 vs control; NS: non-significant vs scrambled siRNA control.

We next investigated the relevance of Piezo1 perturbation on high glucose induced insulin release. As expected, high glucose (17.8 mM for 1 hour) stimulated insulin release (Fig. 5A). Similarly, Yoda1 (10 μM for 1 hour) stimulated insulin release in low glucose buffer (Fig. 5A). The effects of high glucose and Yoda1 were not additive which may suggest either a common underlying mechanism or a limited pool of available insulin (Fig. 5A). Dooku1 alone did not affect insulin release but pre-treatment with Dooku1 (10 μM for 15 min) significantly reduced the effect of Yoda1 (Fig. 5A). The inactive Yoda1 analogue (2e; 10 μM for 1 hour) did not induce insulin release. As expected, exposure to high glucose (17.8 mM for 30 min) increased basal Ca^2+^ level in INS-1 cells (Fig. 5B,C). High glucose also enhanced Yoda1 induced Ca^2+^ influx (Fig. 5D,E). To ascertain that Yoda1 stimulated insulin release is through its action on Piezo1, we tested the effect of Yoda1 on INS-1 cells after knocking down Piezo1 expression using siRNA. Piezo1-specific siRNA transfected cells showed a 78% reduction in Piezo1 mRNA expression compared to the scrambled (control) siRNA transfected cells (Fig. 6A). Importantly, Piezo1 knockdown significantly reduced both Yoda1- and hypotonicity-induced insulin release (Fig. 6B). However, it did not significantly inhibit high glucose-induced insulin release (Fig. 6B).

### Piezo1 agonist regulates insulin release from primary mouse islets

**Figure 7:**
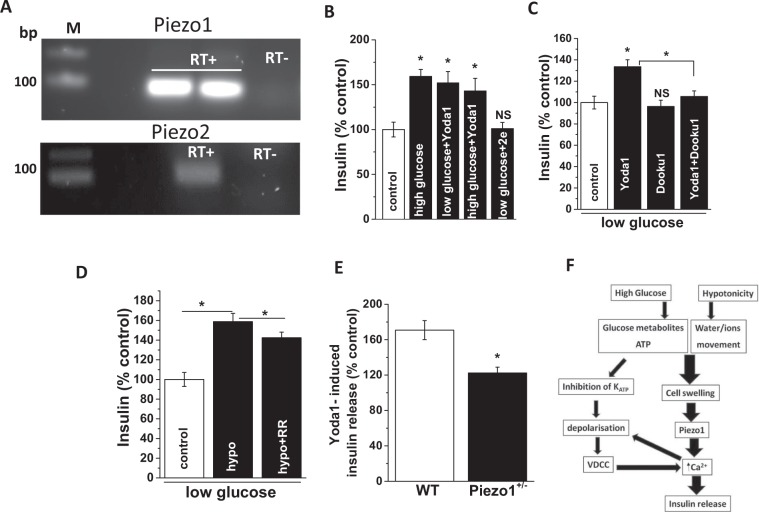
Piezo1 agonist regulates insulin release from pancreatic islets. (**A)** Representative agarose gel electrophoresis picture showing single band at 104 bp (Piezo1) and 101 bp (Piezo2) mRNA expression (RT: with (+) and without (−) reverse transcriptase; bp: base pairs; M: marker) in mouse islets. (**B)** Mean data showing insulin levels in the extracellular solution upon treatment of mouse pancreatic islets with shown chemicals for 1 hour; 2e: non-functional Yoda1 analogue. (**C)** As of (**B**) but showing the effect of Yoda1 with and without Dooku1 pre-treatment. (**D)** Mean data showing insulin levels in the extracellular buffer upon treatment of pancreatic islets with hypotonic buffer for 1 hour in the presence or absence of ruthenium red (RR). (**E)** Mean data showing insulin levels in the extracellular buffer upon 10 μM Yoda1 treatment of wild type (WT) or Piezo1^+/−^ pancreatic islets normalised to respective islets treated with DMSO (controls; 100% represents no effect of Yoda1 on insulin release). (**F)** Schematic flowchart showing possible mechanisms underlying Piezo1 mediated regulation of insulin secretion. Insulin levels were measured by ELISA, normalised against control treatments and expressed as a percentage (%) of control. For Dooku1/RR inhibition experiments, cells were pre-treated with Dooku1/RR for 15 min before the addition of Yoda1/exposure to SS. Data are expressed as mean ± sem; * denotes p < 0.05 vs control; NS: non-significant vs control; $ denotes p < 0.05 vs Yoda1.

To test whether Piezo1 stimulation leads to insulin release from primary β-cells we turned to isolated mouse pancreatic islets. The islets showed expression of both Piezo1 and Piezo2 mRNA (Fig. 7A). Similar to INS-1 cells, high glucose and Yoda1 (10 μM for 1 hour) induced insulin release and the effects were not additive (Fig. 7B). The inactive Yoda1 analogue (2e; 10 μM for 1 hour) had no effect (Fig. 7B). When islets were pre-treated with Dooku1, the effect of Yoda1 was prevented (Fig. 7C). RR produced mild inhibition of hypotonicity induced insulin release from the mouse pancreatic islets as well (Fig. 7D). To further investigate whether Yoda1 induces insulin release by activating Piezo1 channels, we measured Yoda1-stimulated insulin release from pancreatic islets isolated from mice that were haploinsufficient for Piezo1 (Piezo1^+/−^). Yoda1 induced significantly lesser insulin release from Piezo1^+/−^ islets compared to wildtype islets (Fig. 7E). These data suggest that Piezo1 channel activation is a mechanism for inducing insulin release.

## Discussion

Ca^2+^ entry and current induced by the Piezo1 agonist Yoda1, together with the observed Piezo1 mRNA expression, suggest the presence of functional Piezo1 channels in two types of β-cell lines – INS-1 and BRIN-BD11. This is further supported by the predicted responses to known chemical inhibitors of Piezo1 and a non-functional Yoda1 analogue (2e) in Ca^2+^ measurement experiments. Moreover, two well characterised Piezo1 activators (i.e. shear stress and Yoda1) also induced significant insulin secretion from both the β-cell lines and primary mouse islets. The stimulatory effects of hypotonicity, shear stress and Yoda1 were lost in the presence of nonspecific Piezo1 inhibitors and specific Yoda1 antagonist. Importantly, INS-1 cells transfected with Piezo1-specific siRNA and pancreatic islets isolated from Piezo1^+/−^ mice showed significantly reduced Yoda1-induced insulin release. Thus, the results support our hypothesis that stimulating mechanosensitive Piezo1 channels by chemical agonist or cell swelling can induce insulin secretion from pancreatic β-cells.

Yoda1 mimics mechanical stimulation and thus facilitates the study of Piezo1 channels without the need for mechanical stimulation, and has no effect on Piezo2 channels^28^. Overexpressed mouse and human Piezo1 channels were originally shown to be activated by Yoda1 with an EC_50_ of 17.1 and 26.6 μM respectively^28^. Native Piezo1 channels too have responded to Yoda1 at low micromolar concentrations^23,25,31^. However, EC_50_ values were shown to be much lower (2.51 μM for stably overexpressed Piezo1 and 0.23 μM for native channels in human umbilical vein endothelial cells) recently^30^. In β-cells, Yoda1 induced a dose dependent increase in intracellular Ca^2+^ with an EC_50_ of 4.54 to 9 µM (Figs 1C and 3C). Furthermore, a non-functional Yoda1 analogue (2e) failed to induce any Ca^2+^ influx. Both non-selective inhibitors (RR & Gd^3+^) and Dooku1 inhibited Yoda1 induced Ca^2+^ influx significantly. The concentration of Yoda1 required to stimulate Ca^2+^ entry and the lack of effect of 2e, the inhibition of Yoda1-mediated effect by removal of extracellular Ca^2+^, the expected effects of Piezo1 inhibitors and the lack of any inhibitory action of the VDCC blocker (nicardipine) indicate that the Ca^2+^ influx observed in our study is predominantly through Piezo1 channels. Of note, higher concentration (>∼20 μM) of Yoda1 solutions turn increasingly opaque. Therefore, the apparent EC_50_ is likely affected by compound insolubility and may not allow meaningful interpretation.

Ca^2+^ elevation upon hypotonic stimulation has already been reported in various β-cell lines and also in both primary mouse and rat pancreatic β-cells^5,11,14^. It was proposed that hypotonic stimulation leads to membrane depolarization because of activation of volume sensitive outwardly rectifying chloride (Cl^−^) channels, which in turn activated VDCC resulting in Ca^2+^ influx and insulin release^4,10^. In agreement, some studies have shown that hypotonically induced Ca^2+^ elevation in rat pancreatic β-cells was nearly abolished by the VDCC blocker nicardipine^5,11^. However, a recent study in HIT clonal cells demonstrated that Cl^−^ channel blockers such as niflumic acid and DIDS failed to inhibit insulin secretion induced by hypotonic stimulation^12^. It has also been shown that hypotonically induced insulin secretion from the HC9 β-cell line is inhibited by the VDCC blockers nitrendipine and calciseptine, but not by the Cl^−^ channel blocker DIDS^7^. Notably, Gd^3+^ suppressed hypotonicity induced Ca^2+^ elevation in rat pancreatic β-cells and a role for stretch activated cation channels was proposed^14^. Hence, it is clear that there is no consensus from pharmacological studies on the exact mechanism of hypotonicity/cell swelling induced insulin release.

Having neither examined the effect of Piezo1 blockade on the hypotonic response in Ca^2+^ entry nor used Cl^−^ channel blockers, we cannot rule either of them out. However, in the light of present data showing potentiation of Yoda1-induced Ca^2+^ entry by hypotonicity and high glucose, we propose the following model: hypotonicity induced cell swelling leads to Piezo1 activation and Ca^2+^ entry which in turn causes membrane depolarisation and VDCC activation for further Ca^2+^ entry (Fig. 7F). Further careful experimentation with VDCC, Cl^−^, K_ATP_ and Piezo1 channel blockers in different combinations should be performed to clearly elucidate the existence and significance of such a pathway. However, hypotonicity induced insulin release, which could be considered as one of the read-outs for intracellular Ca^2+^ elevation is significantly, but only partially, inhibited by Piezo1 blockade (Figs 4E, 5G, 6B and 7D). Hence both pathways (Cl^−^ mediated and Piezo1/Ca^2+^ mediated depolarisation upon cell swelling) may be participating in hypotonicity induced insulin release. Interestingly, mouse Piezo1 channels are permeable to Cl^−^ as well with a Cl^−^ to Na^+^ permeability ratio (P_Cl_/P_Na_) of 0.14^32^.

Induction of β-cells to produce/release insulin using secretagogues (e.g. sulfonylureas which are K_ATP_ channel blockers) is a clinically used strategy to manage diabetes mellitus^33^. However, current secretagogue pharmaceuticals are not always favoured due to their adverse cardiovascular reactions and reported induction of β-cell apoptosis^34^. The search for non-pharmacological approaches led to the identification of ultrasound as a Ca^2+^ dependent insulin secretion inducer^35,36^. Stimulation of stretch activated channels was proposed as the underlying mechanism. Interestingly, Piezo1 can be activated by ultrasound^37^. Though the significance of Piezo1 in physiological insulin secretion is not evident from our study, using mechanosensitive Piezo1 induction as a strategy to induce insulin secretion has clear clinical potential and warrants further investigation^8^. It is an attractive approach particularly in neonatal diabetes where the disease is caused by gain-of-function mutations in K_ATP_ channels^38^. Hence future experiments using β-cell specific Piezo1 knockout mice are needed to clearly elucidate the role of Piezo1 in the physiological function of β-cells.

## Materials and Methods

### Chemicals and solutions

Unless stated otherwise, all commercially available chemicals were obtained from Sigma‐Aldrich. Stocks of chemicals were reconstituted in DMSO and stored at −20 °C unless stated otherwise. Fura‐2‐AM (Molecular Probes) was dissolved at 1 mM concentration. Pluronic acid F‐127 was stored at room temperature at 10% w.v^−1^ in DMSO. Yoda1 (Tocris) was stored at 10 mM. Yoda1 antagonist (Dooku1) and non-functional Yoda1 analogue (2e) were synthesized and purified and prepared as 10 mM stock solutions^30^. Standard bath solution (SBS) contained (in mM): 140 NaCl, 5 KCl, 1.2 MgCl_2_, 1.5 CaCl_2_, 8 glucose and 10 HEPES titrated to pH 7.4 using NaOH. For zero Ca^2+^ experiments, CaCl_2_ was omitted and mannitol was added to maintain the molarity. For low/high glucose experiments, SBS contained (in mM): 85 NaCl, 5 KCl, 1.2 MgCl_2_, 1.5 CaCl_2_, 2.8/17.8 glucose, 10 HEPES and 100/85 D-mannitol titrated to pH 7.4 using NaOH. For hypo/normotonic experiments, SBS contained (in mM): 85 NaCl, 5 KCl, 1.2 MgCl_2_, 1.5 CaCl_2_, 2.8 glucose, 10 HEPES and 0/100 Mannitol titrated to pH 7.4 using NaOH. Krebs-Ringer bicarbonate HEPES (KRBH) buffer contained (in mM): 120 NaCl, 1.25 KH_2_PO_4_, 1.25 MgSO_4_, 2.68 CaCl_2_, 5.26 NaHCO_3_ and 10 HEPES. Zero-, low- and high- glucose KRBH contained 0, 2.8, 17.8 mM glucose. Osmolality was maintained across all three buffers by adding an equivalent amount of D-mannitol to zero and low glucose KRBH.

### Cell culture

INS-1 832/13 (referred as INS-1) cells derived from rat insulinoma were cultured in RPMI 1640-GlutaMAX-I (Gibco, USA) medium containing 10% heat-inactivated foetal calf serum, penicillin (100 U/ml), streptomycin (100 μg/ml), 1 mM sodium pyruvate, 50 μM 2-mercaptoethanol and 10 mM HEPES. BRIN-BD11 cells derived from rat pancreatic islets were grown in RPMI 1640 -GlutaMAX-I (Gibco, USA) medium containing 10% heat-inactivated foetal calf serum, penicillin (100 U/ml), streptomycin (100 μg/ml). Cultures were maintained at 37 °C under 5% CO_2_ and a humidified atmosphere. Subcultures were established once every 3–4 days by trypsin/EDTA treatment.

### Heterozygous Piezo1 knockout mice

All animal use was authorized by the University of Leeds Animal Ethics Committee and the Home Office, UK. The generation of Piezo1 knockout line was described previously^22^. Mice of age 12–16 weeks were used.

### Mouse pancreatic islet isolation

Islets were isolated following the published protocol^39,40^. Briefly, mice were sacrificed and the pancreata harvested in sterile HEPES buffer. In a sterile hood, the pancreas was washed twice with phosphate buffer saline (PBS) supplemented with 1% penicillin (100 U/ml), streptomycin (100 μg/ml). The pancreas was then minced thoroughly in a glass petri dish and digested using 0.1% collagenase-IV (Sigma) prepared in serum-free RPMI1640 media. Digestion was carried out for 5–7 minutes at 37 °C under 5% CO_2_ with frequent shaking; 2 ml of fetal bovine serum was added to neutralize the collagenase. The digested tissue was then centrifuged at 800 rpm for 10 minutes. The resulting pellet was resuspended in DMEM medium containing 10% foetal calf serum, penicillin (100 U/ml), streptomycin (100 μg/ml) and seeded in a 60 mm Petri dish and cultured for 48 hours at 37 °C under 5% CO_2_ and a humidified atmosphere. C57BL/6 mice aged 2–5 months were used to isolate pancreas, which was conducted in accordance with accepted standards of humane animal care under United Kingdom Home Office Project license No. P606320FB.

### RT-PCR and siRNA

Cells or islets were lysed using TRIzol reagent (Life Technologies), and total RNA was isolated according the manufacturer’s protocol. After DNaseI treatment, RNA was quantified spectrometrically and 1 μg of RNA was reverse transcribed using OligodT primers (Life Technologies) following the manufacturer’s protocol. PCR was performed using *Piezo1* and *Piezo2* specific intron spanning primers (mouse Piezo1: Forward- 5′CTGGACCAGTTTCTGGGACAA3′ and reverse- 5′AGCCTGGTGGTGTTAAAGATGT3′; rat Piezo1: Forward- 5′TTCTTCGGGGTGGAGAGGTA3′ and reverse- 5′TGTCACCATGTGGTTAAGGATG3′; mouse Piezo2: Forward- 5′ATGTGCGTTCCGGTACAATGG3′ and reverse- 5′TGTGTCCTTGCATCGTTGCT3′; rat Piezo2: Forward- 5′GTCCCACCCAATGACTACTATG3′ and reverse- 5′GGACAGGTCTGGGTTTACTATG3′). PCR products were sequenced for confirmation of the products and resolved electrophoretically on a 1.5% agarose gel for presentation purposes. For short interfering (si) RNA transfection studies, INS-1 cells (1 × 10^6^) were grown, pelleted and resuspended in SE Cell Line 4D-Nucleofector solution (Lonza, Switzerland) and mixed with siRNA (rat Piezo1 specific siRNA – sense sequence: 5′GGUCCUAUCUGGAUAUGCUtt3′ or control scrambled siRNA; Ambion) to achieve 30 nM siRNA concentration. The mixture was transferred into a cuvette for electroporation. Cells were then transferred from cuvettes to pre-warmed culture medium and incubated in a 5% CO_2_ incubator at 37 °C. RNA measurements and insulin induction analysis were made after 48 hr. Quantitative expression analysis were performed with SYBR Green based real-time PCR on a LightCycler 480 (Roche, UK) system as described before^22^.

### Intracellular Ca^2+^ measurements

INS-1 or BRIN-BD11 cells were plated in poly‐D‐lysine coated 96‐well plates (Corning, NY, USA) to 80–90% confluence 24 hr before experiments. Prior to recordings, cells were incubated for 1 hr at 37 °C in 2.5 µM fura-2AM dispersed in SBS. The cells were washed for 0.25 hr in SBS at room temperature. Measurements were made on a 96-well bench-top scanning fluorimeter (FlexStation III) with SoftMax Pro v5.4.5 (Molecular Devices, Sunnyvale, CA, USA). Intracellular Ca^2+^ was indicated as the ratio of fura‐2 emission (510 nm) intensities for 340 and 380 nm excitation. Experiments were performed at room temperature (21 ± 2 °C). For Piezo1 inhibition, hypotonicity and high glucose experiments, the inhibitors/modified bath solutions were added on to the cells at the wash point (after fura-2 loading) and the same concentrations were maintained throughout the experiment. When showing agonist-induced changes in fluorescence we have performed baseline subtraction, calculated the mean of all data points from 3.5 to 4.5 minutes after the addition of agonist and presented the results as ΔCa^2+^ (ΔF380/340). To show the change in baseline Ca^2+^, the mean was calculated from all raw fluorescence data points before the addition of agonist.

### Electrophysiology

Conventional whole cell patch-clamp recordings were performed at room temperature as previously described^22^. Patch pipette solution contained (in mM): 145 CsCl, 2 MgCl_2_, 10 Hepes, 1 EGTA (free acid), 5 ATP (sodium salt), and 0.1 Na·GTP (sodium salt), titrated to pH 7.2 with CsOH. The extracellular solution was SBS. Signals were amplified with an Axopatch 200B patch clamp amplifier and pCLAMP10 software (Molecular Devices). A ramp-voltage protocol from –100 mV to +100 mV of 1 s in duration was applied every 10 s from a holding potential of 0 mV. Current signals were filtered at 1 kHz and analyzed off-line using Clampfit software (version 10.2, Molecular Devices). Patch pipettes fabricated from borosilicate glass capillaries had resistances of 2–4 MΩ. Cells were plated 24 hr previously on glass coverslips at 20–30% confluence.

### Insulin secretion stimulation and assessment

Cells or islets were washed with zero glucose KRBH buffer for 1 hr at 37 °C. Dooku1 pre-treatment was achieved by replacing the wash buffer with fresh buffer containing 10 μM Dooku1 during the last 15 min of the wash period. After washing, fresh low glucose KRBH buffer containing test chemicals (Yoda1 and the analogues) was added onto the cells/islets and incubated for 1 hr at 37 °C. High glucose KRBH and hypotonic SBS were used for measuring high glucose and hypotonicity induced insulin release. Cells were exposed to circular shear stress by placing the culture plates on an orbital rotating platform (Grant Instruments) housed inside the incubator for 1 hr. The radius of culture wells was 10 mm and the rotation rate was set to 210 rpm, which generated swirling motion of medium around the edges of the wells producing tangential shear stress. The shear stress on the cells was calculated as 12 dyne/cm^2^ ^41^. The supernatant was collected and stored at −80 °C until analysis. RR pre-treatment was done by replacing the wash buffer with fresh buffer containing 30 μM RR during last 15 min of the wash period. For islets, buffer change and supernatant collection was performed by centrifuging the islets at 800 rpm for 10 min. Insulin levels were determined using an ELISA kit according to the manufacturer’s protocol (from Crystal Chem and RayBio for mouse and rat respectively). For islets, the insulin level was normalised to the total protein of each sample. Protein quantification was carried out by the BCA assay (Pierce) after lysing the islets in the protein lysis buffer containing (in mM), 50 HEPES, 120 NaCl, 1 MgCl_2_, 1 CaCl_2_, 10 NaP_2_O_7_, 20 NaF, 1 EDTA, 10% glycerol, 1% NP40, 2 sodium orthovanadate, 0.5 µg/mL leupeptin, 0.2 PMSF, and 0.5 µg/mL aprotinin (Invitrogen Cell Extraction Buffer). For analysis of the insulin release data, all samples’ data were normalised to the control in that particular experiment considering the control as 100%.

### Data analysis and statistics

All data were analysed using Origin 2016 software. Results are expressed as mean ± SEM of at least 3 independent repeats. Comparisons within groups were made using paired Students t-tests and between groups using unpaired Students t-test, as appropriate; for multiple comparisons ANOVA with Bonferroni’s correction was performed; p < 0.05 was considered statistically significant.

## Data Availability

The datasets obtained during the current study are available from the corresponding author on reasonable request.
